# Vibration-Induced Nystagmus in Patients with Ménière’s Disease: Is There a Correlation to Endolymphatic Hydrops?

**DOI:** 10.3390/audiolres15050125

**Published:** 2025-09-28

**Authors:** Joan Lorente-Piera, Melissa Blanco, Raquel Manrique-Huarte, Adriana David, Victor Suarez-Vega, Angel Batuecas-Caletrío, Gloria Liaño Esteve, Pablo Dominguez, Nicolás Pérez-Fernández

**Affiliations:** 1 Department of Otorhinolaryngology, Clínica Universidad de Navarra, 31008 Pamplona, Spain; joanlopi5@gmail.com (J.L.-P.); rmanrique@unav.es (R.M.-H.); 2 Department of Otorhinolaryngology, Clínica Universidad de Navarra, 28027 Madrid, Spain; nperezfer@unav.es; 3 Department of Otorhinolaryngology, Hospital Universitario General de Villalba, 28400 Madrid, Spain; adridavid6@gmail.com; 4 Department of Radiology, Clínica Universidad de Navarra, 28027 Madrid, Spain; vvega@unav.es (V.S.-V.); glianoestes@unav.es (G.L.E.);; 5 Department of Otorhinolaryngology, Complejo Asistencial Universitario de Salamanca, University of Salamanca, 37008 Salamanca, Spain; abatuc@usal.es

**Keywords:** Ménière disease, vibration induced nystagmus, endolymphatic hydrops, perilymphatic enhancement, vestibular herniation

## Abstract

**Background/Objectives**: Skull vibration-induced nystagmus (SVIN) is a rapid bedside test that reveals vestibular asymmetry. Its clinical utility in Ménière’s disease (MD) remains controversial, particularly regarding its association with radiological endolymphatic hydrops (EH). This study aimed to evaluate the relationship between SVIN, audiovestibular parameters, and EH severity in patients with unilateral definite MD. **Methods**: This prospective observational study was conducted at a tertiary academic referral center and included patients with unilateral MD who underwent SVIN testing (SVT), audiovestibular evaluation (PTA, cVEMP, oVEMP, vHIT, and caloric testing), and 3T MRI with gadolinium-enhanced 3D-FLAIR sequences to quantify EH. **Results**: In total, 84 patients were included in the study. SVIN was present in 57.14% of patients (n = 48), with ipsilesional nystagmus being the most frequent subtype (64.58%). Patients with SVIN had significantly higher vestibular EH (*p* = 0.017) and vestibular endolymphatic ratio (REL) in the affected ear (*p* = 0.019). Disease duration (*p* = 0.026) and shorter time since last vertigo spell (*p* = 0.018) were also associated with SVIN presence. REL correlated moderately with disease duration (r = 0.390, *p* < 0.001), PTA (r = 0.576, *p* < 0.001), and number of vertigo spells (r = 0.236, *p* = 0.031), but not with time since last crisis (r = −0.127, *p* = 0.252). ROC analysis yielded an AUC of 0.735 for REL in predicting SVIN. **Conclusions**: SVIN correlates with the severity of vestibular EH. This finding indicates a stimulus-locked response of a vestibular asymmetry rather than a purely structural alteration.

## 1. Introduction

Skull vibration-induced nystagmus (SVIN) is a simple bedside test for detecting vestibular asymmetry. Applying a 100 Hz bone-conducted vibration to either mastoid rapidly produces a mainly horizontal nystagmus with three key traits: it starts instantly with stimulation, usually beats toward the healthy ear in unilateral vestibular loss, and stops as soon as the vibration ceases [1,2].

SVIN is a mainly otolithic response as these receptors act as seismometers at high frequencies and accelerometers at low ones and the pattern of response results from simultaneous stimulation of canal afferents in both labyrinths [3]. It is extremely useful in the clinical setting such as vestibular neuritis [4], after intratympanic gentamicin treatment [5], and non-operated vestibular schwannoma [6], and has helped characterize responses in superior semicircular canal dehiscence [7,8]. SVIN test (SVT) results in Meniere’s disease (MD) are inconsistent, with positive responses reported in 28% to 71% of patients depending on diagnostic criteria. When SVIN beats toward the healthy ear, it often indicates greater caloric hypofunction and appears in 15% to 63% of cases [9,10].

This variability may be related to the evolving course of Ménière’s disease (MD), which is well defined by the changing pattern of spontaneous nystagmus (SN). Accordingly, two clinical phases can be distinguished: the paretic phase, characterized by a spontaneous peripheral vestibular nystagmus of the inhibitory type, beating toward the unaffected (contralesional) side [11]; and the irritative phase, in which the SN reverses direction and beats toward the affected (ipsilesional) side, often representing a recovery nystagmus [12,13]. These phases shift over time and are closely related to the interval since the last vertigo episode. This temporal factor may also account for variability in other vestibular tests, including the vestibulo-ocular reflex, post–head-shake nystagmus, and even the caloric test [14,15,16].

Due to the demonstrated usefulness of MRI in the diagnosis of endolymphatic hydrops (EH) in MD, efforts to relate the different diagnostic tests with the objectification of hydrops in MRI are constant [17,18,19]

The aim of this study is to analyze the findings in the SVT in patients with unilateral MD and to correlate the finding to the degree of auditory and vestibular deficit and to the degree of EH as detected in the MRI. Given the limited specificity of current functional tests in detecting the structural correlate of vestibular hydrops, our study addresses a critical gap. By integrating functional and structural perspectives, we aim to position SVIN as a potential phase-sensitive biomarker, capable of capturing the dynamic vestibular asymmetry associated with Ménière’s disease rather than a static indicator of uncompensated loss.

## 2. Materials and Methods

### 2.1. Patients, Inclusion Criteria and Exclusion Criteria

The Research Ethics Committee of the University of Navarra (project number 2021.199) has approved this study. All patients included in this study have provided explicit consent for the use of their data for research purposes and written informed consent was obtained from all subjects. We included patients older than 18 years, diagnosed with unilateral MD, and, according to common criteria, in category definite [20]; none of them had previously been treated with either intratympanic gentamicin or inner ear surgery.

All patients underwent audiological and vestibular testing on the same day. MRI was performed either the same day or within 48 h in the majority of cases, and in all instances within a maximum interval of 14 days. This narrow window was chosen to minimize fluctuations inherent to MD, although we acknowledge that functional and radiological changes may occur even within <24 h of a vertigo episode. Therefore, all functional and radiological data were collected within a two-week window following confirmation of definite unilateral MD. Diagnosis, tests and follow-up were conducted by four senior authors (NPF, RMH, MBP and ABC), while the imaging study was conducted by three expert radiologists (PD, VSV and GLE).

Exclusion criteria: Presence of spontaneous nystagmus at the time of evaluation, bilateral MD, significant psychiatric comorbidities likely to impair informed consent, data reliability or protocol adherence and refusal or inability to provide written informed consent or to participate in the study.

### 2.2. Nystagmus Evaluation

All patients included had normal otoscopy and normal eye motility: saccades and smooth pursuit. Spontaneous and gaze evoked nystagmus (with and without visual fixation) and the SVT were recorded.

The SVIN was evoked in the sitting position by applying a handheld vibrator (VVIB 100; Synapsys, Marseille, France) at 100 Hz with a peak-to-peak amplitude of 0.5 mm to each mastoid separately. Each stimulation lasted 15 s and was repeated at least twice per side to ensure reproducibility. No dorsal neck stimulation was performed.

Nystagmus evoked upon stimulation was recorded using videonystagmography goggles (VF405, Interacoustics, Assens, Denmark) in complete darkness, with subjects instructed to maintain straight-ahead gaze during stimulation. SVIN was considered positive when a directionally consistent nystagmus appeared during both right and left mastoid stimulation, with no directional dissociation. The nystagmus had to be present throughout the stimulation period, with an abrupt onset and offset strictly time-locked to the stimulus. Although quantitative slow-phase velocity (SPV) data were not collected systematically, positivity required a visible slow-phase movement exceeding >2°/s on videonystagmography.

All procedures were performed under real-time clinical supervision by trained personnel, with immediate access to medical support if needed. Patients were continuously monitored for dizziness, nausea, or imbalance, and stimulation was promptly interrupted in cases of discomfort. No adverse events were recorded, and all participants tolerated the test without complications. The procedure was conducted in accordance with institutional safety standards and ethical guidelines.

### 2.3. Examination and Complementary Tests

The audiovestibular tests included pure tone audiometry (PTA) (AC40, Interacoustics), auditory and vestibular evoked myogenic potentials (VEMP, Eclipse Interacoustics, Denmark), Video Head Impulse Test (vHIT, ICS^®^ Impulse, Natus Medical, Middleton, WI, USA) and the caloric test (VisualEyes™505, Interacoustics, Middlefart, Denmark).

- Audiological Evaluation. Findings were reported in terms of PTA thresholds from 0.5 to 4 kHz (mean between 0.5 Hz, 1 kHz, 2 kHz y 4 kH), expressed in decibels hearing level (dB HL). The audiometric evaluation was performed during the quiescent phase, not during the fluctuating phase.
- Vestibular Evaluation. vHIT was used to analyze the gain of the vestibulo-ocular reflex, establishing a cutoff for normality at ≥0.8, and also to evaluate the presence or absence of corrective saccades, both covert and overt types. For VEMP, both cervical (cVEMP) and ocular (oVEMP) tests were conducted. An abnormal vestibular function was defined as a VEMP response in both ears with an interaural asymmetry ratio (IAAR %) exceeding 40% [21] Burst tones of 500 Hz were used for monaural auditory stimulation using previously calibrated ABR3A inserted hearing aids. The intensity of the acoustic stimulus was 97 dB normalized hearing level. A Blackman envelope was configured (rise/fall time 2 ms, plateau time 0 ms). 100 averages were presented at a rate of 5.1/s. The response evoked by cVEMP describes a positive (p13) and negative (n23) wave. In oVEMP, the response presents a negative (n10) and positive (p16) wave. IAAR was calculated according the following formula:
  $$\mathrm{IAAR}=\frac{unaffected\;ear\;amplitude-affected\;ear\;amplitude}{unaffected\;ear\;amplitude+affected\;ear\;amplitude}\;\times \;100.$$
  The caloric test was performed using water irrigation at two standard temperatures (44 °C and 30 °C), with each ear tested separately. The resulting responses were analyzed based on the SPV of the induced nystagmus. Jongkees’ formula was applied to calculate canal paresis (considered normal when <21%) [22].
- Imaging studies: All MR studies were performed at 3 Tesla magnets, either a Magnetom Vida or a Magnetom Skyra (Siemens Healthineers, Erlangen, Germany), with 20-channel and 32-channel phased-array receiver coils, respectively. A single dose of intravenous paramagnetic contrast agent gadobutrol (0.1 mmoL/mL, Gadovist, Bayer AG, Zurich, Switzerland) was administrated at a dose of 0.1 mL per kg of body weight. Images were acquired 4 h after the administration of contrast. We performed quantitative and qualitative evaluations of EH.

The volumetric measurement of the entire vestibule was performed semi-automatically with the T2 SPACE cisternography sequence, and vestibular endolymph volume was determined using the 3D REAL-IR sequence. All measurements were performed in advanced visualization software Siemens Syngo.via, version VB50B (Siemens Healthineers) by a single neuroradiologist with more than 7 years of experience on hydrops imaging, who used the pencil tool to manually outline anatomical boundaries on each sequence. For each patient, four volumes were obtained: right and left vestibular and endolymph volumes, respectively.

A releative measure (REL) was obtained by dividing the endolymphatic volume (calculated in the 3D-REAL-IR sequence) by the total vestibule volume (calculated in the T2 cisternography sequence) and multiplying by 100. The volumes were thus expressed in cm^3^ and the relative volume of endolymph to the vestibule in percentage (%). In previous work we determined the relative volume of endolymph in the affected ear (RELAFF) of patients with unilateral MD that separates patients with moderate-severe (radiologically significant) EH and those with mild or no EH using segmentation trees. A 60% RELAFF cut-off yielded a sensitivity of 88.7% and specificity of 82.1%. The ROC curve for this threshold showed an AUC of 0.903, indicating strong discrimination between significant and non-significant EH [23].

The degree of cochlear hydrops (CEH) was determined using a three-level scale ranging from 0 to 2 (normal, mild, and severe), in an axial section passing through the modiolus and encompassing the greatest portion of the cochlea. A four-level scale was used for the assessment of vestibular hydrops (VEH), the optimal visualization plane being the one displaying the greatest anatomical extent of the vestibule (normal, mild, moderate and severe). Perilymphatic enhancement (PE) and endolymphatic herniation (EHern) were also analyzed.

### 2.4. Statistical Analysis

Given the non-normal distribution of most continuous variables (assessed with the Shapiro–Wilk test), non-parametric methods were used for descriptive comparisons. To evaluate the association between SVIN status and radiological variables, we performed multivariable binary logistic regression models, adjusting for clinically relevant covariates including disease duration, time from the last vertigo episode, and age at imaging. Radiological results were analyzed separately for the SVIN-positive and SVIN-negative subgroups to explore differential patterns between groups. Even more, to find correlation between clinical variables and REL, a Spearman correlation test was performed.

For continuous metrics such as the REL, group-wise differences were assessed with Mann–Whitney U tests, and with Wilcoxon signed-rank tests for paired comparisons between affected and non-affected ears when bilateral data were available. Receiver operating characteristic (ROC) curve analyses were also performed to determine the ability of REL in the affected ear to discriminate between SVIN-positive and SVIN-negative cases. The significance of each predictor within the multivariable models was assessed using Wald tests, and the overall model fit was evaluated through likelihood ratio tests (LRT) by comparing the full model against the intercept-only model.

All statistical analyses were conducted using GraphPad Prism v8.0.1 (GraphPad Software Inc., San Diego, CA, USA). To account for multiple comparisons in univariate analyses, a Bonferroni correction was applied, reducing the risk of inflated type I errors, keeping a two-sided significance threshold set at *p* < 0.05.

## 3. Results

### 3.1. Population: Clinical and Demographic Data

A total of 84 patients were included in the study. Clinical and demographic data are summarized in Table 1. Group comparisons were made between SVIN-positive and SVIN-negative patients. All *p*-values presented in Table 1 refer to differences between these two groups.

Patients with SVIN had a shorter disease duration on average (*p* = 0.026) and experienced their last vertigo episode more recently (*p* = 0.018). However, no significant differences were observed when analyzing each variable and its implication in the development of different types of nystagmus (ipsilesional, contralesional, or vertical) in the subgroup analyses.

### 3.2. Analysis of Nystagmus Patterns Neurotological Findings

Among the 84 patients included in the study, 48 (57.14%) showed a positive SVIN response, while it was absent in the remaining 36 patients (42.86%). Among those with a positive SVIN (n = 48), the most common pattern was an ipsilesional nystagmus, observed in 64.58% (n = 31), followed by a contralesional nystagmus in 18.75% (n = 9), and a vertical nystagmus in 16.67% (n = 8). The chi-square test revealed statistically significant differences between the three directions of nystagmus (χ^2^ = 40.3, *p* < 0.001).

### 3.3. Radiological Parameters

**Figure 1:**
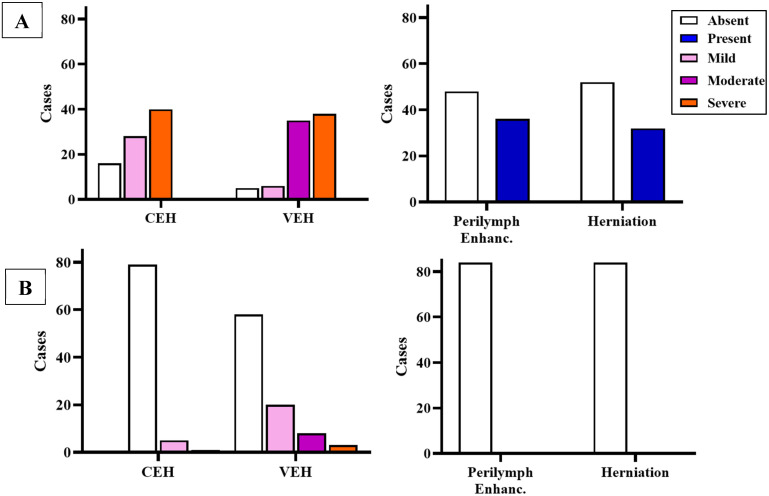
Distribution of radiological findings in the affected ear (**A**) as well as the unaffected ear (**B**).

In the affected ear, all patients exhibited EH in either the cochlear or vestibular compartments (Figure 1A). VEH was more prevalent, observed in 92.86% of cases (n = 78), compared to CEH, which was present in 80.95% (n = 68). In contrast, in the non-affected ear, only 5 patients (5.95%) presented CEH, whereas 30.95% (n = 26) showed some degree of VEH (Figure 1B).

As previously described, binary logistic regression analyses were performed to assess the association between each radiological parameter and the presence of SVIN. The results of these models, including odds ratios (ORs), 95% confidence intervals (CIs), and *p*-values, are summarized in Table 2 and Table 3.

In the affected ear, both a higher REL and the severity of VEH were significantly associated with positive SVIN. In contrast, CEH, perilymphatic enhancement, and endolymphatic herniation did not show significant associations. In the non-affected ear, none of the radiological variables—including REL—showed statistically significant associations with SVIN, although mild grades of cochlear hydrops were still exclusively observed in SVIN-positive patients. After performing a Spearman correlation to assess the relationship between clinical variables and REL, we found that it correlated moderately with disease duration (r = 0.390, *p* < 0.001), PTA (r = 0.576, *p* < 0.001), and the number of vertigo spells (r = 0.236, *p* = 0.031), but not with time since the last crisis (r = −0.127, *p* = 0.252) or with Tumarkin crises (r = 0.046, *p* = 0.482).

**Figure 2:**
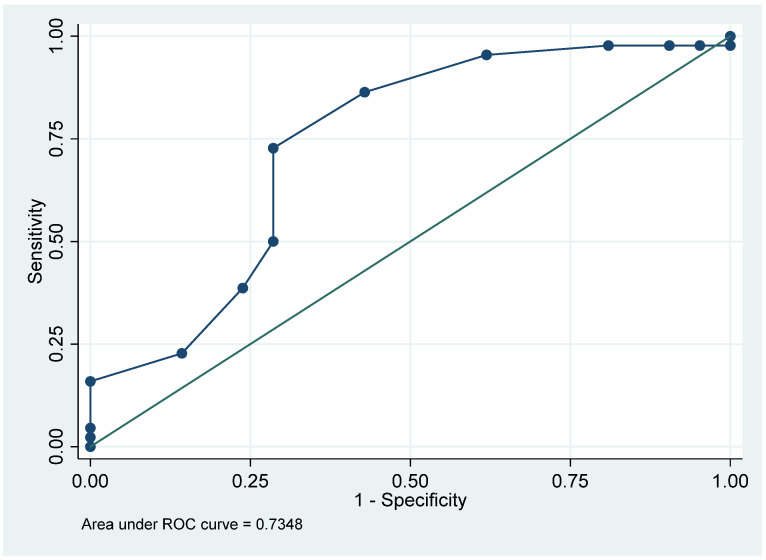
ROC curve evaluating the ability of the REL value in the affected ear to discriminate between patients with and without SVIN.

Finally, we performed a ROC curve analysis to assess the ability of RELAFF value to discriminate between patients with and without a positive SVIN. The area under the curve (AUC) was 0.735, indicating a moderate discriminatory power according to standard statistical interpretation. Based on the curve, the optimal cutoff point—defined as the value maximizing the Youden index—was 0.740, providing the best trade-off between sensitivity and specificity (Figure 2).

### 3.4. Audiovestibular Outcomes

**Figure 3:**
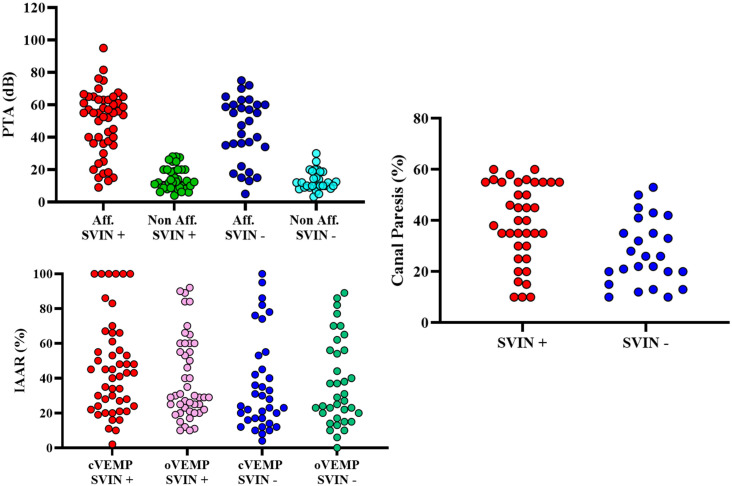
Jitter plots showing distribution of audiovestibular test results across patients. Y-axis units are shown for each parameter: pure-tone average (PTA, dB) cVEMP and oVEMP interaural asymmetry ratio (IAAR, %), and canal paresis (%).

Table 4 and Figure 3 summarize the audiovestibular findings and the results of the logistic regression analyses assessing their association with SVIN. As expected, PTA showed a clear asymmetry between affected and non-affected ears (47.39 ± 19.07 dB vs. 14.47 ± 6.98 dB), but no significant differences were observed between SVIN-positive and SVIN-negative groups for either ear. The IAAR of cVEMP and oVEMP showed wide variability across groups, reflecting the heterogeneity of vestibular dysfunction, but no significant associations with SVIN were found. Abnormal vHIT responses were more frequent in SVIN-positive patients (33.3% vs. 8.3%), showing a statistical trend toward significance (OR = 3.56; 95% CI = 0.94–13.43; *p* = 0.059). Similarly, caloric responses—analyzed as continuous canal paresis values (36.63 ± 15.85% in SVIN+ vs. 27.32 ± 14.68% in SVIN−; *p* = 0.878) or using the ≥21% pathological threshold—did not differ significantly between groups.

## 4. Discussion

Our research shows that in unilateral MD patients, SVIN occurs more often with longer disease duration and recent vertigo episodes, typically beats toward the affected side, and correlates with EH severity, signaling the shift from moderate to severe vestibular hydrops. A key contribution of this study is the demonstration that a simple bedside test such as SVT can be directly correlated with quantitative imaging metrics of hydrops. This functional–radiological linkage highlights the role of SVT as a non-invasive, phase-sensitive biomarker, capable of reflecting real-time vestibular asymmetry in relation to structural pathology.

The presence of SVIN is a valuable indicator of vestibular asymmetry between the two ears, particularly when assessing semicircular canal function. However, it is less sensitive to detecting asymmetries in otolith organ function as measured in our study [24,25]. In Ménière’s disease, the fluctuating and often incomplete dysfunction creates a more complex scenario. Unlike acute or stable vestibular lesions, the degree of asymmetry in MD can vary from day to day, influenced by changes in endolymphatic pressure and the interval since the last vertigo episode [13]. In this context SVIN should be interpreted as a stimulus-locked response reflecting the functional asymmetry present at the time of testing, rather than definitive evidence of chronic uncompensated loss. In Ménière’s disease, this asymmetry is dynamic, fluctuating with endolymphatic pressure and the temporal proximity to vertigo attacks. Consequently, SVIN positivity reflects a state of phase-dependent imbalance, which may or may not persist over time [24,25,26].

In our cohort, SVIN was observed in 57.14% of cases. Nonetheless, no single audiovestibular test—including vHIT, cVEMP, oVEMP, PTA or caloric paresis—significantly predicted SVIN presence in univariate logistic regression models. While our data collection was temporally restricted to a maximum two-week interval, we acknowledge that fluctuations in both vestibular asymmetry and hydrops expression may occur over much shorter timeframes in MD. The association between SVIN presence and time since last vertigo episode further underscores this variability. Ideally, future studies should implement synchronized or longitudinal protocols that monitor both SVIN and radiological markers (such as RELAFF or VEH) across multiple timepoints, especially in or very close (>24 h) to crisis, to better capture the dynamic interplay between structural and functional asymmetries.

Our results partially align with previous literature indicating that in MD, the presence of SVIN may not necessarily reflect a stable vestibular deficit [26,27]. Instead, it could reflect a stimulus-driven asymmetry in vestibular input, consistent with the immediate onset and cessation of nystagmus strictly time-locked to vibration, as previously described in experimental and clinical studies [1,3]. This interpretation is supported by our findings: the absence of a strong predictive correlation between individual vestibular tests and SVIN occurrence suggests a mismatch between peripheral end-organ dysfunction and the global asymmetry required to elicit SVIN. This is different to the situation found in stable unilateral damage as in vestibular neuritis [4,28] or after intratympanic treatment also in MD [29]. Another possible explanation lies in the varying responsiveness of the inner ear to vibratory stimuli across different disease stages, potentially influenced by the extent and frequency-dependent effects of endolymphatic hydrops.

In our cohort, 64.58% of SVIN-positive patients exhibited nystagmus beating toward the affected side, which aligns with previous literature in unilateral vestibulopathy. However, this contrasts slightly with our previous findings reported by Marques-Santos et al. [13], who described a prevalence of approximately 45% for this phenomenon and noted absence of nystagmus in only 25% of cases. Nevertheless, the fact that our study excluded patients with spontaneous nystagmus, while up to 50% of those in the afore mentioned work exhibited it, may also help explain these discrepancies. On the other hand, we also noticed a small subset showed reversed polarity, with nystagmus beating toward the non-affected ear despite documented EH. Several hypotheses may account for this. One is the possibility of paradoxical hyperfunction of the hydropic ear during early stages of EH expansion. Prior studies have noted that in MD, unlike other stable vestibulopathies, SVIN direction can be inconsistent and susceptible to short-term variation [24,25,26].

Previous investigations have explored specific correlations between the severity of endolymphatic hydrops and a range of audiovestibular assessments [23,30,31]. Although establishing a direct correlation between radiological and audiovestibular findings was not the principal objective of the present study, such associations may nonetheless yield meaningful contextual insights. The clinical dissociation between audiometric and vestibular findings is noteworthy [32,33]. As previous works of our group has demonstrated [34], RELAFF usually shows a strong correlation with PTA, but not with cVEMP or oVEMP responses, suggesting a functional decoupling between cochlear and vestibular deterioration. This phenomenon may be attributed to compartment-specific patterns of endolymphatic hydrops progression [35] or to the inherent resistance of vestibular sensory epithelia to hydropic pressure [36].

Although abnormal vHIT responses were more frequent in SVIN-positive patients (33.33% vs. 8.33%), this association did not reach statistical significance. This trend suggests that reduced horizontal canal gain may contribute to the generation of vibration-induced nystagmus, although other factors—including the fluctuating nature of vestibular function in Menière’s disease—likely modulate the response. Similarly, cVEMP and oVEMP asymmetry ratios (IAAR) showed wide interindividual variability and no significant difference between SVIN-positive and SVIN-negative patients. Rather than interpreting this as a null result, the absence of correlation is better explained by the frequency- and receptor-specific domains of each test: SVIN captures high-frequency vestibular asymmetry driven mainly by canal afferents; caloric irrigation probes ultra–low-frequency canal function; and VEMPs reflect otolithic pathways. Therefore, the lack of association is consistent with the complementary physiology of these assessments rather than a contradiction. This overall pattern supports the notion that SVIN is predominantly a canal-driven phenomenon, with otolithic pathways playing a less consistent role. These findings align with electrophysiological and clinical studies demonstrating that vibration preferentially activates irregular afferents from the semicircular canals while simultaneously recruiting, to a lesser extent, otolithic afferents [24]. Consequently, SVIN in this cohort reflects the combined but predominantly canal-mediated response to high-frequency vibration, which may be variably expressed depending on the degree and dynamics of the peripheral asymmetry present at the time of testing. Other methods of testing for otolithic function (ocular counter-rolling) will probably help to better understand its role.

A key contribution of this study is the analysis of REL, a continuous radiological metric derived from volumetric sequences such as the T2 SPACE and 3D-REAL-IR that quantifies the volumetric proportion of EH. Although qualitative assessment remains the most widely used approach, our group advocates for a quantitative evaluation with the RELAFF as the main parameter, since our identified cut-off values have shown good correlation with qualitative scales. In a previous volumetric study, a 60% ELR threshold yielded a sensitivity of 88.7% and specificity of 82.1% for detecting moderate-to-severe EH, with an AUC of 0.9037, suggesting strong discriminative power. Notably, this value closely approximates the optimal REL cut-off of 74.38% identified in the current cohort for SVIN prediction. In our regression models, both VEH and REL in the affected ear emerged as significant predictors of SVIN, underscoring their relevance in identifying structural alterations of the inner ear. However, despite the strong anatomical signal, RELAFF alone does not fully explain the presence of SVIN. The observation that some patients with marked EH do not exhibit SVIN supports the hypothesis that nystagmus arises only when vestibular asymmetry surpasses a critical threshold, suggesting that structural changes, while necessary, are not sufficient on their own. Nonetheless, substantial hydropic accumulation within the vestibular endolymphatic space may still play a pivotal role in facilitating this imbalance. While VEH and REL in the affected ear are significantly associated with SVIN, other radiological markers—such as endolymphatic herniation, typically seen in more advanced stages, or early perilymphatic enhancement patterns [37]—do not show consistent correlations. These findings point to a multifactorial mechanism in which SVIN reflects not just static anatomical disruption, but also the dynamic interplay between structural pathology and vestibular functional asymmetry.

Finally, the diagnostic value of SVIN in MD remains bounded by the fluctuating nature of the disease. In contrast to other inner ear conditions characterized by stable, permanent impairment, MD is marked by dynamic and variable functional changes. The presence of SVIN may fluctuate depending not only on the current hydropic load but also on the patient’s compensation status. Nonetheless, the high proportion of SVIN beating toward the affected side, together with its partial association with RELAFF and clinical parameters, supports its utility in characterizing functional asymmetry in selected patients. Its low cost, simplicity, and non-invasiveness continue to position it as a useful adjunctive tool in the vestibular clinic.

### Limitations

This study has several limitations that should be acknowledged. First, although the sample size of 84 patients is relatively robust for a single-center study, the cross-sectional design limits the ability to infer causality or assess temporal fluctuations in SVIN expression, which is particularly relevant in a disease known for its episodic nature. Second, the absence of quantitative analysis of nystagmus SPV prevents correlation between the intensity of SVIN and the degree of vestibular or radiological impairment. This limits the functional resolution of the test and may obscure subtler associations. Third, although the time interval between SVT and MRI was short in all cases, the absence of a truly simultaneous evaluation may have limited our ability to capture transient fluctuations in hydrops expression and vestibular responsiveness. Moreover, by excluding patients with spontaneous nystagmus, we may have overlooked a subset in which SVIN behavior is influenced by underlying baseline activity. Future research should include these patients and prioritize longitudinal, time-resolved protocols. Lastly, the heterogeneity and limited predictive power of audiovestibular tests in this context highlight the complex interplay between anatomical, functional, and central compensatory mechanisms, which may require multimodal or dynamic testing paradigms for comprehensive characterization.

## 5. Conclusions

To sum up, this study demonstrates that SVIN is a valuable functional marker for detecting vestibular asymmetry in patients with unilateral Menière’s disease. The significant associations observed between SVIN and radiological parameters, particularly RELAFF and VEH, highlight the relevance of vestibular hydrops in shaping the vibratory response. These findings suggest that SVIN reflects a dynamic state of functional asymmetry, driven by the interaction between structural changes and fluctuating vestibular function, rather than a static anatomical alteration alone. Thus, beyond its value as a clinical bedside sign, SVIN emerges as a complementary biomarker to MRI, with the potential to provide a rapid, non-invasive, and phase-sensitive readout of vestibular imbalance in patients with Ménière’s disease.

Importantly, the absence of SVIN in some patients with advanced hydrops, as well as the lack of consistent correlations with conventional functional tests, indicates that the generation of SVIN likely requires surpassing a specific threshold of asymmetry. While we did not find robust predictors of nystagmus directionality, the observed variability reinforces the need for future longitudinal studies to assess the temporal relationship between disease stage, hydropic load, and the polarity of the induced nystagmus.

## Figures and Tables

**Table 1:** Demographic, clinical, and radiological variables obtained in patients included in the cohort. SD: Standard deviation. * *p* < 0.05.

Clinical and Demographic Data		*p*-Value
**N (Men: Women)**	50:34 patients	0.093
**Age (Mean ± SD)**	52.60 ± 10.86 years	0.314
**Disease duration (Mean ± SD)**	5.27 ± 6.52 years	0.026 *
**Duration < 3 years/>3 years**	42:42 patients	N/A
**Affected side (Right/Left)**	51:33 ears	0.462
**Days since last dizzy spell (Mean ± SD)**	33.12 ± 64.40 days	0.018 *
**Number of dizzy spells last six months (Mean ± SD)**	6.60 ± 4.70 spells	0.445
**Tumarkin crises last six months (Cases, %)**	11 patients (13.95%)	0.534

**Table 2:** Radiological variables in the affected MD ear, for SVIN positive and negative groups * *p* < 0.05.

MRI Evaluation of the Affected Ear		SVIN + (n = 48)	SVIN − (n = 36)	*p* Value	OR (CI 95%)
**Cochlear EH (CEH)**	None	8 (16.67%)	8 (22.22%)	0.655	1.26 (0.52–3.09)
	Mild	16 (33.33%)	11 (30.56%)		
	Severe	24 (50.00%)	17 (47.22%)		
**Vestibular EH (VEH)**	None	2 (4.17%)	4 (11.11%)	0.017 *	3.15 (1.23–8.07)
	Mild	1 (2.02%)	6 (16.67%)		
	Moderate	21 (43.75%)	13 (36.11%)		
	Severe	24 (50.00%)	13 (36.11%)		
**Vestibular E. Ratio (REL)**	Affected (RELAFF)	80.11 ± 10.27%	64.13 ± 13.30%	0.019 *	7.99 (1.41–45.22)
**Perilymphatic** **enhancement (PE)**	Absent	19 (39.58%)	14 (38.89%)	0.989	0.91 (0.36–2.30)
	Present	29 (60.41%)	22 (61.11%)		
**Endolympatic** **herniation (EHern)**	Absent	29 (60.41%)	24 (66.67%)	0.646	1.27 (0.50–3.23)
	Present	19 (39.58%)	12 (33.33%)		

**Table 3:** Radiological variables in the non-affected MD ear, for SVIN positive and negative groups.

MRI Evaluation of the Non-Affected Ear		SVIN + (n = 48)	SVIN − (n = 36)	*p* Value	OR (CI 95%)
**Cochlear EH (CEH)**	None	44 (91.67%)	35 (97.22%)	0.285	0.55 (0.19–1.56)
	Mild	4 (8.33%)	1 (2.78%)		
	Severe	0 (0.00%)	0 (47.22%)		
**Vestibular EH (VEH)**	None	35 (72.92%)	23 (63.88%)	0.461	0.65 (0.25–1.68)
	Mild	10 (20.83%)	11 (30.56%)		
	Moderate	3 (6.25%)	2 (5.56%)		
	Severe	0 (0.00%)	0 (0.00%)		
**Vestibular E. Ratio (REL)**	Non-affected (RELNAFF)	38.12 ± 14.36%	35.13 ± 12.03%	0.349	5.43 (0.16–187.51)
**Perilymphatic enhancement (PE)**	Absent	48 (100%)	36 (100%)	-	-
**Endolympatic herniation (EHern)**	Absent	48 (100%)	36 (100%)	-	-

**Table 4:** Summary of audiovestibular test results in the study population.

Audiovestibular Tests		SVIN + (n = 48)	SVIN − (n = 36)	*p*-Value	OR (CI 95%)
**Hearing**					
**PTA**	Affected ear	47.39 dB ± 19.07	44.52 ± 20.01 dB	0.315	1.01 (0.99–1.04)
	Non-affected ear	14.47 dB ± 6.98 dB	13.06 ± 6.02 dB	0.206	1.04 (0.98–1.11)
**vHIT**					
	Normal	32 (66.67%)	33 (91.67%)	0.122	0.38 (0.11–1.25)
	Pathologic	16 (33.33%)	3 (8.33%)	0.059	3.56 (0.94–13.43)
**VEMPs**					
**Ocular**	Present/Absent	38/10	30/6	0.355	0.99 (0.98–1.01)
	IAAR (%)	37.24 ± 27.18	31.40 ± 27.76		
**Cervical**	Present/Absent	42/6	31/5	0.481	1.01 (0.99–1.02)
	IAAR (%)	39.40 ± 30.70	34.41 ± 31.66		
**Caloric Test**					
	Canal Paresis	36.63 ± 15.85	27.32 ± 14.68	0.878	0.97 (0.93–1.01)

## Data Availability

The data pertaining to this study can be shared upon request to the corresponding author.
